# An Overview of NCA-Based Algorithms for Transcriptional Regulatory Network Inference

**DOI:** 10.3390/microarrays4040596

**Published:** 2015-11-16

**Authors:** Xu Wang, Mustafa Alshawaqfeh, Xuan Dang, Bilal Wajid, Amina Noor, Marwa Qaraqe, Erchin Serpedin

**Affiliations:** 1 Department of Electrical and Computer Engineering, Texas A&M University, College Station, TX 77843, USA; E-Mails: xu.wang@tamu.edu (X.W.); mustafa.shawaqfeh@tamu.edu (M.A.); xuandt89@tamu.edu (X.D.); bilalwajidabbas@tamu.edu (B.W.); marwa@tamu.edu (M.Q.); 2 Institute of Genomic Medicine, University of California San Diego, La Jolla, CA 92093, USA; E-Mail: amnoor@ucsd.edu

**Keywords:** gene, transcription factor, transcriptional regulatory network, network component analysis

## Abstract

In systems biology, the regulation of gene expressions involves a complex network of regulators. Transcription factors (TFs) represent an important component of this network: they are proteins that control which genes are turned on or off in the genome by binding to specific DNA sequences. Transcription regulatory networks (TRNs) describe gene expressions as a function of regulatory inputs specified by interactions between proteins and DNA. A complete understanding of TRNs helps to predict a variety of biological processes and to diagnose, characterize and eventually develop more efficient therapies. Recent advances in biological high-throughput technologies, such as DNA microarray data and next-generation sequence (NGS) data, have made the inference of transcription factor activities (TFAs) and TF-gene regulations possible. Network component analysis (NCA) represents an efficient computational framework for TRN inference from the information provided by microarrays, ChIP-on-chip and the prior information about TF-gene regulation. However, NCA suffers from several shortcomings. Recently, several algorithms based on the NCA framework have been proposed to overcome these shortcomings. This paper first overviews the computational principles behind NCA, and then, it surveys the state-of-the-art NCA-based algorithms proposed in the literature for TRN reconstruction.

## 1. Introduction

For every soccer team, the coach is responsible for directing the team to victory. The primary aim is to score as many goals as possible and, at the same time, thwart the other team from doing the same. The coach may choose some players over others. Even among team members, some players attack, others defend, whereas some are good as half-back players. Moreover, the core players that form the playing team do not remain the same throughout the game. Keeping in mind the dynamics of the game, the coach may direct some players to replace others, ensuring the primary aim of the game, to win, remains intact. In close comparison with this framework, the cell does not operate very differently. The coach of the cell, the DNA within the nucleus, directs different team members, TF and genes, to execute cellular functions and complex biological processes, which help the cell to adapt to varying dynamics, including external stimuli, as well as internal changes. The team members, TFs and genes, work together to express or suppress different metabolic pathways at different instances of the cell’s life. Particularly, these TFs contain DNA binding domains that allow them to bind to specific regions of DNA, called promoters [1]. By binding to these promoters, TFs initiate the process of converting genes into proteins. Transcription factor activities (TFAs) refer not only to the connectivity of any particular TF, but also to its level of activity. The connectivity of a particular TF informs its team members to collaborate in order to regulate RNA polymerase, which in its turn controls in terms of expressing or suppressing genes. TFAs cannot be measured directly; rather, they can be inferred from gene expression data. Furthermore, TRN represent interactions between genes and TFAs within a cell and offer a global perspective in the cellular behavior. Understanding the structure of TRNs and estimating TFAs provide insight into the cellular dynamics present in healthy and diseased tissues and organs and hold the potential to help in diagnosing, characterizing and determining cures for various diseases [2].

In the literature, several computational frameworks have been proposed to analyze regulatory interactions, which are briefly summarized below. The first class models the TRN as a dynamic system. Particularly, [3] and [4] describe gene expression as a linear and continuous time first-order differential equation. On the other hand, Boolean network models [5,6] quantize gene expressions by only two discrete levels: ON and OFF. The expression level of each gene is the Boolean function of the expression levels of other genes. These methods are generally performed using a small number of time series data and, thus, lead to an under-determined problem [7]. Another approach for TRN reconstruction is referred to as the co-expression (or relevance) networks, in which two genes are connected if the similarity between them exceeds a predefined threshold. Examples of similarity measures used in constructing relevance networks include correlation [8] and mutual information [9,10]. Relevance networks are helpful to understand the fundamental topological features of biological networks, but they do not infer causal relations among genes. The algorithms falling into the third category are commonly described as probabilistic graphical models [11,12,13], which include Gaussian graphical models (GGMs) and Bayesian networks (BNs). In GGMs, the network or graph is constructed based on the notion of conditional independence, and two genes are connected if and only if they are independent given the expression levels of all other genes. GGMs are formulated using undirected graphs and represent an example of full conditional models, since the conditional dependency is considered with respect to all other genes. On the other hand, BNs entail directed acyclic graphs, and the conditional dependency is measured with respect to all subsets of the other genes [11,14]. One limitation of probabilistic graphic models is that they have strong assumptions on the joint distribution that prevent representing or interpreting some biological relationships. For example, cyclic graphs are not allowed in the BN framework. In this way, it ignores self-feedback loops among genes that are natural features in genetic networks. Additionally, the applications of probabilistic graphic models are generally limited to the network with the number of experimental measurements significantly larger than the number of genes, since analyzing the structure of large-scaled genetic networks using probabilistic graphic models is highly complex. Besides dynamic models, co-expression networks and probabilistic graphical models, structural equation modeling (SEM) also represents a widely-used technique for TRN inference [15,16]. Generally, an SEM consists of a structural model and a measurement model. The structural model describes the causal relations between the latent variables, while the measurement model depicts the relations between latent variables and observed measurements.

**Figure 1:**
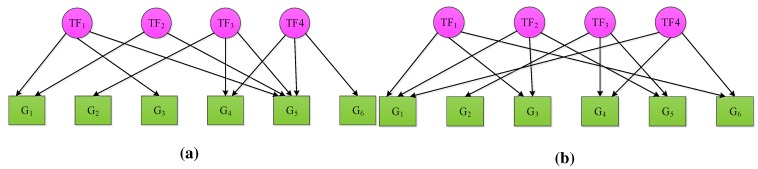
Examples of two transcription regulatory networks (TRNs) with six genes and four transcription factors (TFs), but different connectivity topologies.

Recently, studies dedicated to TRN inference using the network component analysis (NCA) technique have begun to emerge in the literature [17]. NCA establishes a parameter estimation problem and reconstructs TRNs following a statistical signal processing viewpoint. Since NCA-based algorithms do not require time series data, they can collect the experimental data from different time intervals and combine them to increase the samples size and prevent the under-determination problem. Even with a limited number of experiments, NCA-based algorithms are still able to reconstruct TRNs with a large number of TFs and genes (See Section 3.3 for more details). Moreover, NCA-based algorithms take advantage of some prior knowledge about the connectivity patterns of the genetic network, which is becoming available via high-throughput experiments [18] or data mining of interaction information [19,20,21]. The assumed mathematical model for NCA is represented by the following system of linear equations [17]:

(1) X=AS+Γ

where $X\in R^{N\times K}$ represents the log ratios of expression values of *N* genes at *K* time points of the microarray dataset, $A\in R^{N\times M}$ denotes the connectivity strength between *N* genes and *M* TFs, $S\in R^{M\times K}$ stands for the activities of *M* TFs at *K* time points and $\Gamma \in R^{N\times K}$ represents the measurement noise. Examples of two TRNs with six genes and four TFs, but different connectivity topologies, are shown in Figure 1.

Generally, in Equation (1), X cannot be uniquely decomposed as the product of two matrices A and S, unless further constraints are imposed. Principal component analysis (PCA) [22] and independent component analysis (ICA) [23] represent two conventional statistical algorithms that can provide valid solutions provided that the input signals present in S are independent and/or orthogonal. However, such an assumption generally does not hold for biological signals in practice. Accounting for this fact, Liao *et al*. [17] proposes NCA, which incorporates the prior information about TF-gene regulation, to infer TRNs. As will be discussed in detail in Section 2, NCA is an iterative computational algorithm that ensures the uniqueness of decomposition solutions. Due to some drawbacks of NCA, such as the stringent conditions required to apply NCA, several alternative NCA-based algorithms have been proposed in the literature to improve NCA from different perspectives, such as less restrictive assumptions, lower computational complexity and higher robustness against noise, outliers and modeling errors.

The rest of the paper, which proposes to provide a review of the major algorithms reported for NCA, is organized as follows. Section 2 introduces the NCA framework and the mathematical details of the NCA algorithm. Extensions of NCA are presented in Section 3. These extensions still rely on the NCA algorithm, but improve the applicability range of NCA by requiring less stringent assumptions. In Section 4, alternative NCA-based algorithms proposed in the literature for TRN inference are surveyed. A few illustrative computer simulation results highlighting the performance of major NCA algorithms are presented in Section 5. In addition, the comparison of these algorithms and some recommendations on how to choose the appropriate algorithm are discussed in Section 6 based on the simulation results in Section 5. Finally, Section 7 summarizes the content of this paper.

## 2. NCA

In the case when both matrices A and S are unknown, the decomposition problem in Equation (1) admits an infinite number of solutions. Fortunately, prior information is becoming available for many biological systems, e.g., ChIP-on-chip (ChIP-on-chip (also known as ChIP-chip) represents a technology that combines chromatin immunoprecipitation (“ChIP”) with a DNA microarray (“chip”)) data indicate whether a certain gene interacts with a certain TF. This prior information is incorporated within NCA mathematically via the constraint A(I)=0, where *I* presents the indices of zero elements in the connectivity matrix A, indicating a certain level of connectivity information. NCA requires three identification criteria to ensure a unique solution up to a scalar ambiguity:

1 The connectivity matrix A must be full-column rank.
2 If a column of A is removed along with all of the rows corresponding to the nonzero entries of the removed column, the remaining matrix must still be full-column rank.
3 The TFA matrix S must have full row rank.

**Figure 2:**
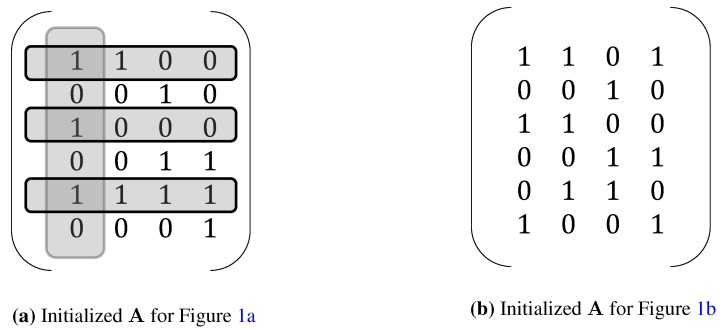
An example of (**a**) a non-identifiable pattern and (**b**) an identifiable pattern.

To test whether the system meets the above-mentioned first two criteria, matrix A must be first initialized based on the prior knowledge available about connectivity. Specifically, $a_{ij}$ is assigned to zero if (i,j)∈I, and it assumes any arbitrary nonzero value otherwise. Once A is initialized, matrix A is tested to see if it presents a full-column rank. Then, we sequentially remove each column of A, as well as the genes connected to the removed TF and test whether the remaining reduced matrix still presents full-column rank. Consider TRNs in Figure 1 as an example. The initialized connectivity matrices for Figure 1a,b are illustrated in Figure 2a,b, respectively. The initialized connectivity matrix in Figure 2a is not identifiable, since the reduced matrix obtained by removing the first column along with the first, third and fifth rows is not full-column rank. This condition violates the second criterion of NCA. The initialized connectivity matrix in Figure 2b, on the other hand, satisfies all three identification criteria. In terms of the third criterion, it cannot be tested *a priori*, but it implies that the number of TFs must be less than or equal to the number of time points, *i.e.*, M≤K. This rank criterion is verified after S is simulated using NCA [17].

**Figure 3:**
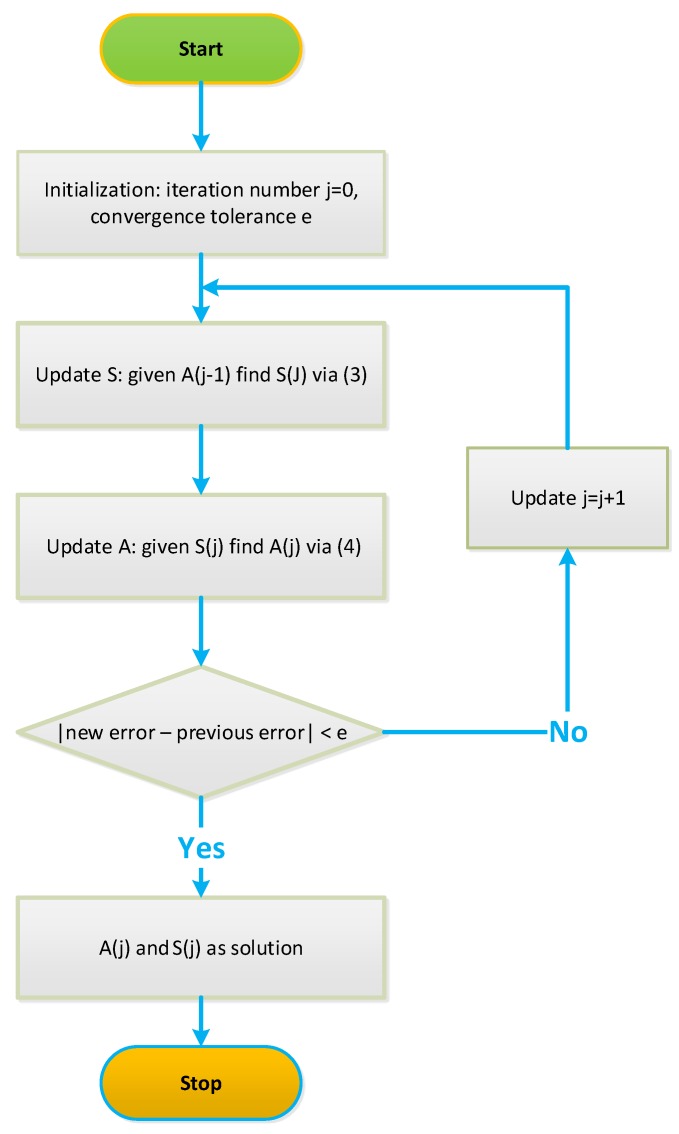
Network component analysis (NCA) algorithm.

NCA aims to solve the following optimization problem:

(2) $$\begin{array}{cc} \min_{A,S} & \;||X-{AS||}_{F}^{2},\\ \mathrm{s.t.} & \;A(I)=0, \end{array}$$

where $\left|\right|\cdot {\left|\right|}_{F}$ denotes the Frobenius norm. NCA employs an alternate least-squares (ALS) approach to iteratively update A and S. At iteration *j*, given A(j−1), *i.e.*, the value of A at iteration (j−1), the estimate of S(j) is obtained by solving the following least-squares (LS) problem:

(3) $$\begin{array}{cc} S(j) & =\arg\min_{S}||X-{A\left(j-1\right)S||}_{F}^{2}\\ \mathrm{s.t.} & \;\;s_{i,j}^{\left(l\right)}\leq s_{i,j}\leq s_{i,j}^{\left(u\right)}, \end{array}$$

where the constraint is included to ensure that the elements of S remain in the domain of biologically-sensitive values [17]. The optimization problem Equation (3) can be solved by standard convex optimization tools, such as the interior point method [24]. Once S(j) is obtained, the next step is to update A(j) via the following optimization problem:

(4) $$\begin{array}{cc} A(j) & =\arg\min_{A}||X-{AS\left(j\right)||}_{F}^{2}\\ \mathrm{s.t.} & \;\;A\left(I\right)=0,\;a_{i,j}^{\left(l\right)}\leq a_{i,j}\leq a_{i,j}^{\left(u\right)}, \end{array}$$

where the constraint $a_{i,j}^{\left(l\right)}\leq a_{i,j}\leq a_{i,j}^{\left(u\right)}$ is also used to constrain the domain of A. Particularly, eliminating the zero elements in A removes the connectivity constraint A(I)=0. This leads to a new least-squares problem with a lesser number of variables, which can also be solved using the same method employed to solve Equation (3). If the decrease in the total least-squares error after updating A is above a preset value *e*, the algorithm keeps running. Otherwise, it stops. A diagram illustrating the operation of the NCA is shown in Figure 3. Simulation results in [17] demonstrate that NCA was successfully applied to the microarray data generated from yeast *Saccharomyces cerevisiae*, and the activities of various TFs during the cell cycle were reconstructed.

## 3. Extensions of NCA

Despite its successful implementation in yeast data, NCA exhibits several shortcomings, which prevent its application to a wide class of regulatory network inference problems. In the literature, several papers have been proposed to tackle these issues. In this section, we focus on several improvements for NCA proposed recently in the literature. In these works, the core estimation methods are identical to NCA, but some enhancements have been implemented to make the NCA algorithm more applicable to various setups.

### 3.1. Motif-Directed NCA

In the original NCA work [17], the prior information about the connectivity matrix, *i.e.*, A(I), is provided by high-throughput experiments. However, the high-throughput ChIP-on-chip data are not available for some common species, such as rodents and humans [25]. With respect to this fact, Wang *et al*. [25] proposed a motif-directed NCA (mNCA) algorithm, which incorporates the motif information to obtain the prior network structure information and to infer TRNs. Due to the fact that the regulation between TFs and genes occurs only after TFs bind to the DNA sequence motifs in the gene’s promoter region [25], the authors incorporate the motif information to recover the interaction between TFs and genes. Moreover, since the prior topology information, either from ChIP-on-chip data or motif analysis, comes from biological experiments, it may contain many false positives/negatives. Thus, a stability analysis is further proposed in [25] to extract stable TFAs from the NCA algorithm. Specifically, the authors of [25] intentionally perturb the connectivity information and use the Pearson correlation coefficient as a stability measurement to determine whether the estimated TFAs are stable or not. Experimental results on muscle regeneration microarray data demonstrate that mNCA is able to reveal important TFAs, as well as their connectivity strength to corresponding genes.

### 3.2. Generalized NCA

The work in [26] proposed the generalized NCA (gNCA) in an attempt to improve the NCA criteria. gNCA extends the system identification criteria required by NCA by additionally incorporating the prior information about regulatory matrix S, such as the regulatory information obtained from regulatory gene knockouts (a gene knockout (KO) refers to a genetic technique through which one or more genes from an organism are made inoperative (“knocked out”)) [26]. Thus, for the gNCA criteria to guarantee a unique decomposition solution, they require a full column rank condition for A, a full row rank condition for S and an additional condition that preserves the essential features of A and S. In this way, given the topology information about S, the uniqueness of the decomposition problem might still be ensured by alternatively checking the gNCA criteria, even if the connectivity structure of A does not satisfy the NCA criteria. Even when the connectivity topology satisfies the NCA criteria, gNCA reduces the number of parameters to be estimated by combining the prior information about S.

### 3.3. Revised NCA

The work in [27] also focuses on enhancing the NCA criteria. The work in [27] proposed revised NCA (NCAr), where the third criterion of NCA is revised to improve the applicability of NCA. As discussed earlier, to ensure a unique solution for the matrix factorization problem, the third criterion of NCA requires the matrix S to have full row rank, which implies that the number of TFs must be less than or equal to the number of experiments. This requirement significantly limits the sample size of TFs. The work in [27] revises the third criterion of NCA based on the observation that most of the genes are only regulated by a smaller number of TFs than the total number of TFs (*i.e.*, the connectivity matrix A is row-wise sparse). In particular, this condition, instead of being associated with the rank properties of matrix S, is related to the rank properties of reduced-size matrices. Particularly, it requires that the number of experiments for each gene be greater than or equal to the number of TFs regulating that gene. The revised criterion enables NCA to be applicable to a wider class of TRN inference problems, since the number of TFs regulating a gene is generally less than five or six [27]. In this way, a large dimensional regulatory network can be uniquely inferred, even in the presence of a limited number of experiments.

### 3.4. Generalized-Framework NCA

The original NCA work requires the biological system to satisfy all three criteria to ensure a unique decomposition up to a scaling factor. However, NCA only checks the compliance for the initialized matrix A. It may occur that the derived matrix A at certain iterations violates the NCA criteria. The work in [28] generalizes the NCA criteria, such that the system identification can be determined directly from the connectivity (topology) information, rather than checking the rank properties of the unknown connectivity matrix A. In other words, if a certain connectivity topology, *i.e.*, $A(I_{0})$, meets the newly-derived conditions, then all matrices $A\in A(I_{0})$ satisfy the first and second criterion of NCA, and thus, they guarantee the feasibility of A during each iteration of the NCA algorithm. To deal with the issue that the connectivity topology does not satisfy the newly-derived conditions or the TF matrix does not satisfy the third criterion of NCA (for example, when M>K, the linear independence of TFs is violated), the authors in [28] alternatively seek to infer subnetworks by removing the selected TF node together with all of its associated genes until all of the system identification criteria of the reduced subnetwork are verified. The resulting algorithm is referred to as generalized-framework NCA (gfNCA) [28].

## 4. Alternative NCA-Based Algorithms

In this section, we review some alternative NCA-based algorithms that were also recently reported in the literature. Different from the algorithms discussed in Section 3, where mNCA, gNCA, NCAr and gfNCA utilize the NCA algorithm to infer TRNs, the algorithms discussed in this section focus on designing more efficient algorithms to estimate the matrices A and S in the NCA system model Equation (1). These algorithms can be roughly classified into two classes, namely the iterative and the non-iterative class.

### 4.1. Iterative NCA Algorithms

As described in Section 2, NCA adopts the ALS approach to iteratively update matrices A and S. Therefore, NCA, along with all of the algorithms that employ ALS, such as mNCA, gNCA, NCAr and gfNCA, is an iterative method. Another example of iterative methods, referred to as robust NCA (ROBNCA), is reviewed next.

#### Robust NCA

ROBNCA [29] is a robust NCA-based approach that tries to cope with the possible noise and outliers present in the microarray data due to erroneous measurements and/or the abnormal response of genes [30]. To counteract the presence of outliers, the system model of TRNs is formulated as:

(5) X=AS+O+Γ

where matrix O models explicitly the presence of outliers. Since typically, only a few outliers exist, the outlier matrix O represents a column-sparse matrix. Accounting for the sparsity of matrix O, ROBNCA aims to solve the following optimization problem:

(6) $$\begin{array}{cc} \left\{\hat{A},\hat{S},\hat{O}\right\} & =\arg\min_{A,S,O}||X-AS-{O||}_{F}^{2}+\lambda_{0}{\left||O|\right|}_{0}\\ \mathrm{s.t.} & \;\;\;A(I)=0, \end{array}$$

where ${\left||O|\right|}_{0}$ denotes the number of nonzero columns in O and $\lambda_{0}$ is a penalization parameter used to control the extent of sparsity of O. Due to the intractability and high complexity of computing the $l_{0}$-norm-based optimization problem, the problem Equation (6) is relaxed to:

(7) $$\begin{array}{cc} \left\{\hat{A},\hat{S},\hat{O}\right\} & =\arg\min_{A,S,O}||X-AS-{O||}_{F}^{2}+\lambda_{2}{\left||O|\right|}_{2,c}\\ \mathrm{s.t.} & \;\;\;A(I)=0 \end{array}$$

where ${\left||O|\right|}_{2,c}$ stands for the column-wise $l_{2}$-norm sum of O, *i.e.*, ${\left||O|\right|}_{2,c}=\sum_{k=1}^{K}\left|\right|o_{k}{\left|\right|}_{2}$, where $o_{k}$ denotes the *k*-th column of O. Since the optimization problem Equation (7) is not jointly convex with respect to {A,S,O}, an iterative algorithm is performed in [29] to optimize Equation (7) with respect to one parameter at a time.

Towards this end, the ROBNCA algorithm at iteration *j* assumes that the values of A and O from iteration (j−1), *i.e.*, A(j−1) and O(j−1), are known. Defining Y(j)=X−O(j−1), the update of S(j) can be calculated by carrying out the optimization problem:

$$S\left(j\right)=\arg\min_{S}||Y\left(j\right)-{A\left(j-1\right)S||}_{F}^{2}$$

which admits a closed-form solution. The next step of ROBNCA at iteration *j* is to update A(j) while fixing O and S to O(j−1) and S(j), respectively. This can be performed via the following optimization problem:

(8) $$\begin{array}{cc} A(j) & =\arg\min_{A}||Y\left(j\right)-{AS\left(j\right)||}_{F}^{2}\;.\\ \mathrm{s.t.} & \;\;\;A(I)=0 \end{array}$$

The problem Equation (8) was also considered in the original NCA paper [17] in which a closed-form solution was not provided. Since this optimization problem has to be conducted at each iteration, a closed-form solution is derived in ROBNCA using the re-parameterization of variables and the Karush–Kuhn–Tucker (KKT) conditions to reduce the computational complexity and improve the convergence speed of the original NCA algorithm. In the last step, the iterative algorithm estimates the outlier matrix O by using the iterates A(j) and S(j) obtained in the previous steps, *i.e.*,

(9) $$O\left(j\right)=\arg\min_{o_{k}}||C\left(j\right)-{O||}_{2}^{2}+\lambda_{2}{\left||O|\right|}_{2,c}$$

where C(j)=X−A(j)S(j). The solution to Equation (9) is obtained by using standard convex optimization techniques, and it can be expressed in a closed form.

It can be observed that at each iteration, the updates of matrices A, S and O all assume a closed-form expression, and it is this aspect that significantly reduces the computational complexity of ROBNCA when compared to the original NCA algorithm. In addition, the term $\lambda_{2}{\left||O|\right|}_{2,c}$ guarantees the robustness of the ROBNCA algorithm against outliers. Simulation results in [29] also show that ROBNCA estimates TFAs and the TF-gene connectivity matrix with a much higher accuracy in terms of normalized mean square error than FastNCA [31] and non-iterative NCA (NINCA) [32], irrespective of varying noise, the level of correlation and outliers.

### 4.2. Non-Iterative NCA Algorithms

This section presents four fundamental non-iterative methods, namely, fast NCA (FastNCA) [31], positive NCA (PosNCA) [33], non-negative NCA (nnNCA) [34] and non-iterative NCA (NINCA) [32]. These algorithms employ the subspace separation principle (SSP) and overcome some drawbacks of the existing iterative NCA algorithms. FastNCA utilizes SSP to preprocess the noise in gene expression data and to estimate the required orthogonal projection matrices. On the other hand, in PosNCA, nnNCA and NINCA, the subspace separation principle is adopted to reformulate the estimation of the connectivity matrix as a convex optimization problem. This convex formulation provides the following benefits: (i) it ensures a global solution; (ii) it allows usage of efficient convex programming techniques, like the interior point method [24]; and (iii) it offers the flexibility of adding additional convex constraints. Since SSP represents the core technique of these non-iterative NCA-based algorithms, this important concept is first explained in the next subsection.

#### 4.2.1. Subspace Separation Principle

Assume matrix X is decomposed into the sum of two other matrices X=B+Γ, where $X\in R^{N\times K}\left(K<N\right)$ stands for the observed data, $B\in R^{N\times K}$ represents the true signal and $\Gamma \in R^{N\times K}$ denotes the noise matrix. SSP attempts to partition the range space of X into two subspaces, where one subspace is spanned by the source signal and the other subspace is spanned by noise. One possible way to do this is via singular value decomposition (SVD). Specifically, the SVD of X takes the form:

(10) $$X=U\Sigma V^{T}=\sum_{i=1}^{K}\sigma_{k}u_{k}v_{k}^{T}\;$$

where the singular values are arranged in a descending order $\sigma_{1}\geq \sigma_{2}\geq ...\geq \sigma_{K}\geq 0$. In the situation where the noise level is low and the signal matrix is not ill-conditioned, the significant singular values (singular values with larger values) correspond to the signal subspace, and the remaining negligible singular values correspond to the noise subspace. Under the assumption of keeping (L<K) singular values as the signal singular values, the SVD of Equation (10) can be decomposed into two components, corresponding to the signal ($X_{L}$) and noise component ($X_{R}$), respectively.

(11) $$X=\underset{X_{L}}{\underset{︸}{U_{L}\Sigma_{L}V_{L}^{T}}}+\underset{X_{R}}{\underset{︸}{U_{R}\Sigma_{R}V_{R}^{T}}}$$

The first term in Equation (11), *i.e.*, $X_{L}$, is called the *L*-rank Eckart–Young–Mirsky (EYM) approximation of X and represents the higher signal-to-noise ratio (SNR) representation of X. Matrix $\Sigma_{L}$ is a diagonal matrix, and it contains the first *L* singular values corresponding to the signal component; and $U_{L}$ and $V_{L}$ correspond to the left and right singular vectors, respectively. Similarly, $\Sigma_{R}$ is a diagonal matrix containing the last K−L singular values corresponding to the noise part, and $U_{R}$ and $V_{R}$ correspond to the left and right noise singular vectors, respectively. Hence, the space of the observed measurements is approximately decomposed into two separate subspaces: signal and noise subspace, respectively. If we still further denote X as the product of the two matrices $A\in R^{N\times M}$ and $S\in R^{M\times K}$, *i.e.*, X=AS+Γ as shown in Equation (1), it is shown in [32] that $U_{R}$ represents a robust approximation of the left null space of A in the case L=M.

#### 4.2.2. FastNCA

FastNCA [31] provides a closed form solution to NCA, and it overcomes in the same time the speed limitations of the original NCA. FastNCA employs a series of matrix partitionings and orthogonal projections to estimate the connectivity matrix on a column-by-column basis. Once matrix A is estimated, matrix S is estimated by a direct application of the least-squares principle:

(12) $$S=A^{†}X\;$$

Next, a detailed explanation of the FastNCA approach to estimate the first column of A, *i.e.*, $a_{1}$, in both the noiseless and noisy case is presented. The same analysis can be repeated for the remaining columns, since the columns in A can be re-ordered by appropriately changing the rows of S.

In the ideal case where no noise exists, the system model in Equation (1) assumes the form:

(13) X=AS

Without loss of generality, the elements in $a_{1}$ are rearranged, such that the nonzero elements are located at the beginning of the vector and the zero elements are placed at the end:

(14) $$a_{1}=\left[\begin{array}{c} {\tilde{a}}_{1}\\ 0 \end{array}\right]\;$$

Then, Equation (13) can be partitioned as:

(15) $$X=\left[\begin{array}{c} X_{c}\\ X_{r} \end{array}\right]=\left[\begin{array}{cc} {\tilde{a}}_{1} & A_{c}\\ 0 & A_{r} \end{array}\right]\left[\begin{array}{c} s_{1}^{T}\\ S_{r} \end{array}\right]=\left[\begin{array}{c} {\tilde{a}}_{1}s_{1}^{T}+A_{c}S_{r}\\ A_{r}S_{r} \end{array}\right]$$

Taking the transpose of Equation (15) results in:

(16) $$X_{c}^{T}=s_{1}{\tilde{a_{1}}}^{T}+S_{r}^{T}A_{c}^{T}$$

(17) $$X_{r}^{T}=S_{r}^{T}A_{c}^{T}\;$$

Extracting ${\tilde{a}}_{1}$ is possible if the term $S_{r}^{T}A_{c}^{T}$ in Equation (16) can be eliminated. This can be determined by using an orthogonal matrix projection. Assuming the orthogonal projection matrix onto $S_{r}^{T}$ is $P_{S_{r}^{T}}^{⊥}$ and multiplying Equation (16) by $P_{S_{r}^{T}}^{⊥}$ leads to:

(18) $$P_{S_{r}^{T}}^{⊥}X_{c}^{T}=P_{S_{r}^{T}}^{⊥}s_{1}{\tilde{a}}_{1}^{T}={\tilde{s}}_{1}{\tilde{a}}_{1}^{T}$$

where ${\tilde{s}}_{1}=P_{S_{r}^{T}}^{⊥}s_{1}$. Therefore, the challenge is to find $P_{S_{r}^{T}}^{⊥}$. From Equation (17), the range space of $S_{r}^{T}$ and the left null space of $X_{r}^{T}$ are the same, since $S_{r}^{T}$ is full column rank (the third NCA criterion). Furthermore, $A_{r}^{T}$ is full row rank (first NCA criterion). Hence, $P_{S_{r}^{T}}^{⊥}=P_{X_{r}^{T}}^{⊥}$. $P_{S_{r}^{T}}^{⊥}X_{c}^{T}$ is known. Therefore, a rank-one factorization of $P_{S_{r}^{T}}^{⊥}X_{c}^{T}$ yields an estimate of ${\tilde{a}}_{1}^{T}$ up to a scalar ambiguity, and it represents the first right singular vector of $P_{S_{r}^{T}}^{⊥}X_{c}^{T}$.

In the noise case, as shown in Equation (1), FastNCA handles the noise in the gene expression measurements by using the concept of subspace separation. This is done by replacing the noisy observation data X with its *L*-rank EYM approximation $X_{L}$ (see Equation (11)). In this way, it follows that:

$$X=U_{L}\Sigma_{L}V_{L}^{T}$$

and moreover:

(19) $$W=U_{L}=XV_{L}\Sigma_{L}^{-1}=\left(AS+\Gamma \right)V_{L}\Sigma_{L}^{-1}=A\tilde{S}+\tilde{\Gamma }$$

where $U_{L}$ is represented by W for simplicity, $\tilde{S}=SV_{L}\Sigma_{L}^{-1}$ and $\tilde{\Gamma }=\Gamma V_{L}\Sigma_{L}^{-1}$.

Partitioning W in the same way as in Equation (15) yields:

(20) $$W=\left[\begin{array}{c} W_{c}\\ W_{r} \end{array}\right]=\left[\begin{array}{cc} {\tilde{a}}_{1} & A_{c}\\ 0 & A_{r} \end{array}\right]\left[\begin{array}{c} {\tilde{s}}_{1}^{T}\\ {\tilde{S}}_{r} \end{array}\right]+\left[\begin{array}{c} {\tilde{\Gamma }}_{c}\\ {\tilde{\Gamma }}_{r} \end{array}\right]$$

which further results in:

(21) $$\begin{array}{cc} W_{c}^{T} & ={\tilde{s}}_{1}{\tilde{a_{1}}}^{T}+{\tilde{S}}_{r}^{T}A_{c}^{T}+{\tilde{\Gamma }}_{c}^{T} \end{array}$$

(22) $$\begin{array}{cc} W_{r}^{T} & ={\tilde{S}}_{r}^{T}A_{r}^{T}+{\tilde{\Gamma }}_{r}^{T} \end{array}$$

Due to noise, a direct repetition of the noiseless case analysis is not applicable, because $P_{{\tilde{S}}_{r}^{T}}^{⊥}\neq P_{W_{r}^{T}}^{⊥}$. The subspace separation principle provides an estimate of $P_{{\tilde{S}}_{r}^{T}}^{⊥}$. Consider the following SVD of $W_{r}$:

(23) $$W_{r}=U_{1}\Sigma_{1}V_{1}^{T}+U_{0}\Sigma_{0}V_{0}^{T}\;$$

where $\Sigma_{1}$ and $\Sigma_{0}$ contain the leading M−1 and last L−M+1 singular values, respectively. Then, an estimate of ${\hat{P}}_{{\tilde{S}}_{r}^{T}}^{⊥}$ is given by:

(24) $${\hat{P}}_{{\tilde{S}}_{r}^{T}}^{⊥}=V_{0}V_{0}^{T}$$

Similar to the noiseless case, ${\tilde{a}}_{1}^{T}$ can be obtained by applying a rank-one factorization for ${\hat{P}}_{{\tilde{S}}_{r}^{T}}^{⊥}W_{c}^{T}$.

#### 4.2.3. Positive NCA, Non-Negative NCA and Non-Iterative NCA

PosNCA [33] modifies the original NCA algorithm in two regards. The first aspect pertains to evaluating matrix A via a convex optimization (instead of ALS, as in the original NCA). The second aspect refers to the addition of the positivity constraints on all of the nonzero elements in the connectivity matrix. This assumption has a biological support [35]. The positivity constraint is valid only in situations where all TFs play the same role (*i.e.*, activating or deactivating) on their corresponding targeted genes. If all of the TFs regulate the genes in a negative way (deactivating), the positivity assumption is maintained by multiplying the activity value in the signal matrix by the value −1. This positivity assumption is a convex constraint, which perfectly integrates with the convex formulation of the problem.

The essence of the formulation of PosNCA as a convex optimization problem relies on the orthogonality between the range space and the left null space. However, the challenge is to find a basis for the left null space of A. Consider C to be a basis for the left null space of A; then, it follows that:

(25) $$C^{T}A=0.$$

In the ideal case (X=AS), the range space and left null space of A are the same as those of X. This is because A is a full column rank (first criterion of NCA) and S is full row rank (third criterion of NCA). Therefore, C is obtained directly from X. In contrast to the noiseless case, there is no direct access to C in the noisy case. Alternatively, SSP provides a robust approximation of C. Consider the SVD $X=U\Sigma V^{T}$, and let U be partitioned as $U=[U_{L},U_{R}]$, where $U_{L}$ is of dimensions N×M and $U_{R}$ is of dimensions N×(N−M). Then, based on the discussion in Section 4.2.1, $U_{R}$ represents an approximation of C ($\hat{C}=U_{R}$). Therefore, A can be estimated by minimizing the Frobenius norm of $\left|\right|{\hat{C}}^{T}A{\left|\right|}_{F}$, while maintaining both constraints, *i.e.*, the structure of the connectivity matrix and the positivity of all nonzero elements in the connectivity matrix. Mathematically, this problem can be formulated as follows:

(26) $$\begin{array}{cc} \hat{A} & =\arg\min_{A}\left|\right|{\hat{C}}^{T}A{\left|\right|}_{F}\;\\ \mathrm{s.t.} & \;\;\;A(I)=0,\;\;\;A(J)\geq c \end{array}$$

where *J* stands for the set of indices of the nonzero elements in A and *c* is small positive constant. The optimization problem in Equation (26) is a convex optimization problem, since both the objective function and constraints are convex. The authors of [33] used an interior point-based method [24] to solve Equation (26). After evaluating A, the signal matrix is estimated using the traditional ALS:

(27) $$S=A^{†}X\;$$

The authors of [34] pioneered nnNCA, which utilizes the separable nature of the estimation problem corresponding to the matrix A in Equation (26) to achieve a computationally-efficient version of their previously-reported algorithm PosNCA. In PosNCA, matrix A is estimated in one shot by solving the optimization problem Equation (26). On the other hand, nnNCA estimates the columns in A in parallel, since each column of the connectivity matrix can be estimated independently of the other columns [34].

Later, NINCA [32] was proposed to further improve the computational efficiency and the estimation accuracy of the framework reported in PosNCA. Analogous to nnNCA, NINCA estimates matrix A on a column-by-column basis. In addition, NINCA does not assume a positive constraint on the non-zero elements of the connectivity matrix and further imposes the constraint $1^{T}\cdot a_{i}=1$ for each column of matrix A to avoid the trivial solution. In terms of the procedure to estimate the TF matrix S, instead of using the traditional least-squares error adopted in [33] and [34], NINCA employs a total least-squares (TLS) algorithm [36] that not only considers the error in S, but also weighs the error in A.

## 5. Simulation Results

In this section, computer simulations are carried out to compare and evaluate the performance of major NCA algorithms. As discussed in Section 3, mNCA, gNCA, NCAr and gfNCA utilize the same estimation method as NCA. Additionally, PosNCA and nnNCA rely on the same framework, *i.e.*, minimizing the Frobenius norm of ${\hat{C}}^{T}A$, where $\hat{C}$ denotes the estimated left null space of the connectivity matrix A and estimating S via the least-squares method. Therefore, only the simulation results pertaining to NCA, FastNCA, ROBNCA, NINCA and PosNCA will be presented in this section.

**Figure 4:**
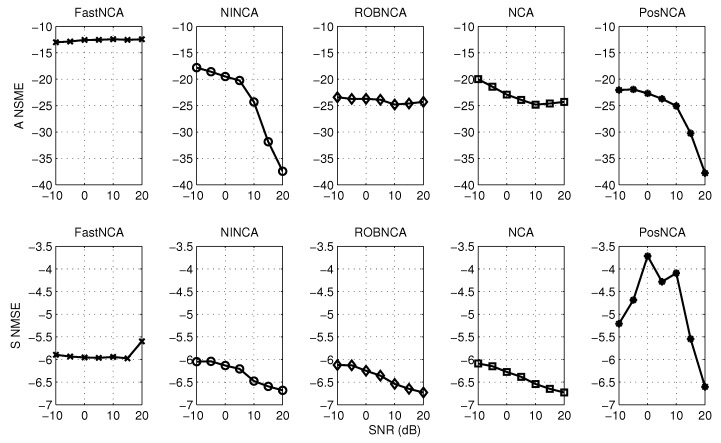
NMSE for different algorithms with respect to SNR from −10 dB–20 dB.

**Figure 5:**
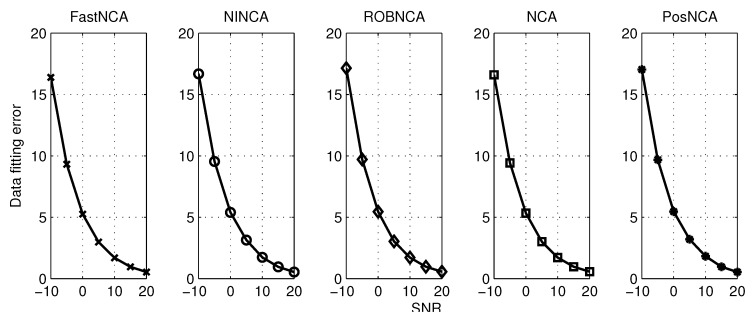
Data fitting error for different algorithms with respect to SNR from −10 dB–20 dB.

**Figure 6:**
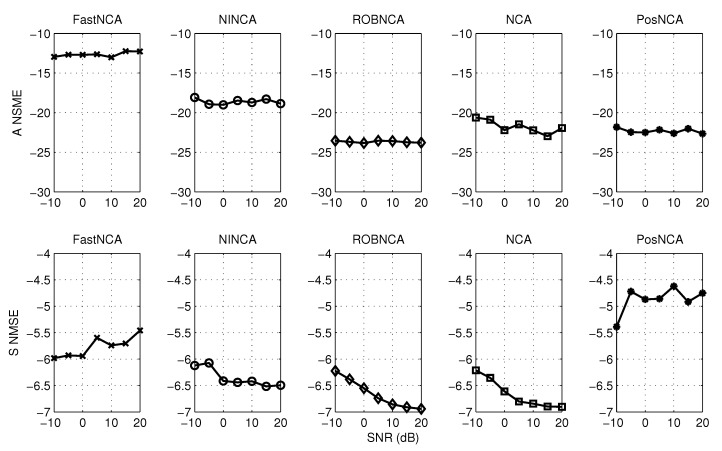
NMSE for different algorithms with respect to SNR from −10 dB–20 dB and outliers with probability 0.1.

**Figure 7:**
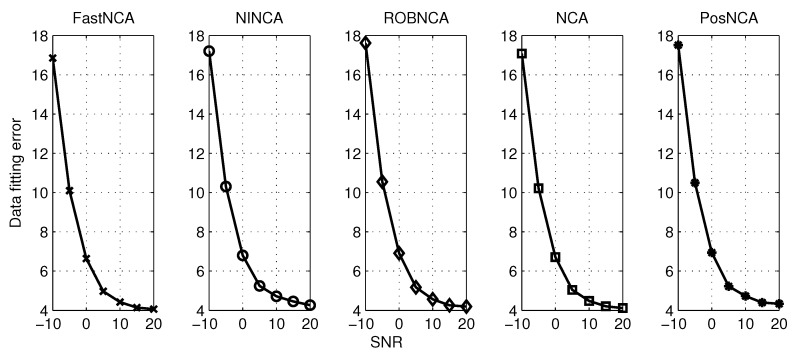
Data fitting error for different algorithms with respect to SNR from −10 dB–20 dB and outliers with probability 0.1.

The synthetic data widely used in [17,29,31,32] are tested. This spectroscopy data contain M=3 hemoglobin solutions obtained by mixing up N=7 pure hemoglobin components, and the absorption spectra consist of K = 300 experiment points, which are measured for wavelengths in the range of 380–700 nm [29]. The aforementioned algorithms are tested when the observed data are corrupted with different levels of Gaussian noise and when the observations contain both Gaussian noise and outliers. The normalized mean square error (NMSE) and the data fitting error (DFE), *i.e.*, $||AS-{X||}_{F}$, are adopted herein to measure the estimation accuracy. The simulation results are averaged over 50 iterations. The algorithms are first simulated by varying the SNR from −10 dB–20 dB. The NMSE for matrices A and S and the DFE are illustrated in Figure 4 and Figure 5, respectively. In terms of the test against both noise and outliers, the outliers are manually added into the observations by modeling them as a Bernoulli process with probability 0.1. The simulation results with respect to NMSE and DFE are depicted in Figure 6 and Figure 7, respectively. Under 10 dB SNR, a comparison of the performance of NCA-based algorithms in both the noise case and noise + outliers case is shown in Table 1. These experiments are performed in MATLAB 7.12.0 with a 2.5-GHz Intel Core i5 processor, and the computation time stands for the average time to perform one iteration of simulation experiments.

**Table 1:** Normalized mean square error (NMSE), data fitting error (DFE) and computation time for different algorithms under 10 dB SNR. NINCA, non-iterative NCA; ROBNCA, robust NCA; PosNCA, positive NCA.

Algorithm	ANSME		SNSME		Data Fitting Error		Computation Time
	Noise	Noise + Outliers	Noise	Noise + Outliers	Noise	Noise + Outliers	
FastNCA	0.0571	0.0500	0.2544	0.2666	1.6973	4.4193	0.0005
NINCA	0.0037	0.0134	0.2250	0.2280	1.7361	4.7164	0.0119
ROBNCA	0.0033	0.0044	0.2218	0.2062	1.7141	4.5630	0.0080
NCA	0.0033	0.0060	0.2217	0.2068	1.7139	4.4809	6.6728
PosNCA	0.0031	0.0055	0.3896	0.3451	1.8200	4.7275	0.2648

**Figure 8:**
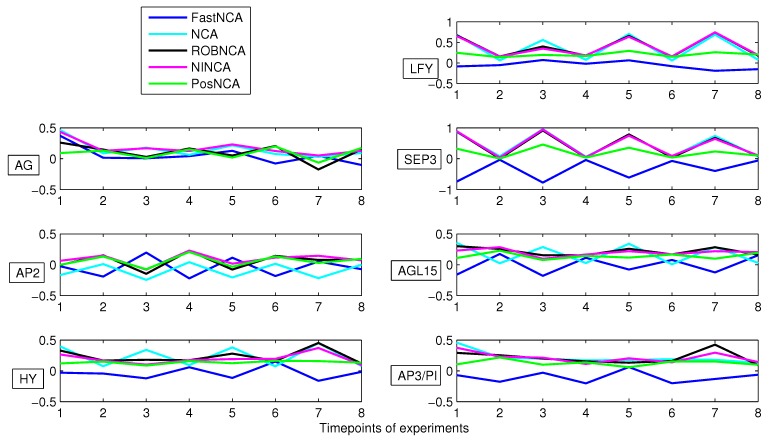
TFA reconstruction: estimation of seven TFAs of the Arabidopsis Gene Regulatory Information Server (AGRIS).

Furthermore, we also employ these algorithms to quantitatively analyze a real dataset. In particular, a plant TRN in floral development using the *Arabidopsis thaliana* dataset housed in the Arabidopsis Gene Regulatory Information Server (AGRIS) [37] is analyzed. The initial dataset consists of 10 TFs and 57 genes. However, only seven TFs, namely LFY, AG, SEP3, AP2, AGL15, HY and AP3/PI, and 55 genes were found to be compliant with the NCA framework [38]. The simulation results to reconstruct the aforementioned seven TFs are depicted in Figure 8. It can be seen that NCA, ROBNCA and NINCA almost obtain an identical estimate for SEP3, and they share a similar trend for the reconstruction of other TFs. On the other hand, with respect to the estimation of SEP3, the results obtained by FastNCA and PosNCA are different from those exhibited by NCA, ROBNCA and NINCA.

## 6. Comparison of NCA-Based Algorithms

Based on the simulation results in Figure 5 and Figure 7, it can be concluded that all algorithms achieve a similar performance in terms of the data fitting error; or roughly speaking, these algorithms obtain a similar estimate of the product of A and S. However, it can be observed from Figure 4 and Figure 6 that different estimation results are obtained when NCA-based algorithms try to separate the product of A and S, respectively. Therefore, in the following subsections, we mainly investigate the simulation performance on estimating the connectivity matrix A and the TF matrix S using the synthetic data. Additionally, some recommendations on how to choose the appropriate algorithm in different scenarios are also discussed.

### 6.1. Estimating the Connectivity Matrix

In terms of performance in the presence of additive noise, Figure 4 depicts that NINCA and PosNCA achieve a higher degree of accuracy when the SNR is high. Moreover, the performance of ROBNCA and NCA is also accurate and consistent compared to FastNCA. When the data are corrupted with both noise and outliers, according to Figure 6, ROBNCA achieves the best performance against outliers. The NSME of NINCA and PosNCA increases significantly compared to the case without outliers, especially when the SNR is high. Even though the performance of FastNCA does not degenerate when outliers exist, the NMSE is still relatively large compared to other algorithms.

### 6.2. Estimating the TF Matrix

Based on the simulation results in Figure 4 and Figure 6, it can be observed that ROBNCA and NCA achieve the minimum NSME in both the noise case and noise + outliers case. Moreover, the existence of outliers does not have an obvious impact on the performance of ROBNCA and NCA. In contrast, the performance of FastNCA and NINCA for estimating the TF matrix S is not robust to outliers. Unlike the good simulation results in estimating A, the performance of PosNCA for estimating S is significantly inferior to all other algorithms. That is probably because PosNCA utilizes the least-squares solution to derive S once obtaining the estimate of A, which is numerically unstable.

### 6.3. Recommendations on Choosing the Appropriate Algorithm

In terms of the average computational time from Table 1, FastNCA is faster than the other four algorithms. However, FastNCA is not recommended herein, since it shows a high degree of inconsistency and inaccuracy. Moreover, even though NCA performs very well in both the noise and noise + outliers cases, the run time of NCA is hundreds and thousands of times slower than the other four algorithms using the small-dimensional synthetic data. It can be inferred that NCA is more computationally inefficient for reconstructing large-dimensional TRNs. In the case where the accuracy of the connectivity matrix is the first priority, PosNCA is recommended due to the fact that PosNCA has a high degree of accuracy in estimating A, especially in the scenario where the SNR is high. NINCA and ROBNCA can be selected as the general methods to solve the TRN inference problem, since they are consistent and accurate in both the noise and noise + outliers cases. Moreover, the run time of NINCA and ROBNCA is also comparable to FastNCA. Between these two algorithms, ROBNCA is more preferable if the existence of outliers is known *a priori*.

## 7. Conclusions

This paper surveys the state-of-the-art NCA-based algorithms proposed in the literature. These algorithms rely on a linear model and concentrate on reconstructing the TFA matrix and the connectivity matrix by using the information provided by microarray gene expression data. The algorithms reviewed herein can be divided broadly into two categories: iterative and non-iterative methods. For the iterative methods, the estimation process for the connectivity matrix and TFA matrix starts with an initial guess, and then, it proceeds through a sequence of iterative steps. The output of each step is fed as an input to the next step. On the other hand, the non-iterative methods aim to overcome the drawbacks of iterative methods, especially to reduce the high computational complexity by reformulating the NCA problem. A summary of the surveyed NCA-based algorithms is illustrated in Table 2 for further details.

**Table 2:** Summary of NCA-based algorithms. mNCA, motif-directed NCA; gNCA, generalized NCA; NCAr, revised NCA; gfNCA, generalized-framework NCA; nnNCA, non-negative NCA; ALS, alternate least-squares; SSP, subspace separation principle; TLS, total least-squares.

Algorithm	Category	Estimation Technique	Contribution
NCA [17]	Iterative	ALS	Proposed the NCA framework and criteria, motivated other NCA algorithms
mNCA [25]	Iterative	ALS	Incorporated motif information to obtain the prior connectivity information
gNCA [26]	Iterative	ALS	Incorporated the prior information about the TFA matrix
NCAr [27]	Iterative	ALS	Revised and extended the third identification criterion
gfNCA [28]	Iterative	ALS	Modified the criteria of NCA, such that they are only related to the prior connectivity information
ROBNCA [29]	Non-iterative	Alternate optimization	Reduced the computational complexity and improved the robustness against outliers
FastNCA [31]	Non-iterative	SSP, rank-1 factorization	Reduced the computational complexity
PosNCA [33]	Non-iterative	SSP, convex optimization	Combined additional prior information to reduce the complexity
nnNCA [34]	Non-iterative	SSP, convex optimization	Combined additional prior information and reduced the complexity
NINCA [32]	Non-iterative	SSP, convex optimization, TLS	Combined additional prior information, reduced the complexity and improved the estimation accuracy
